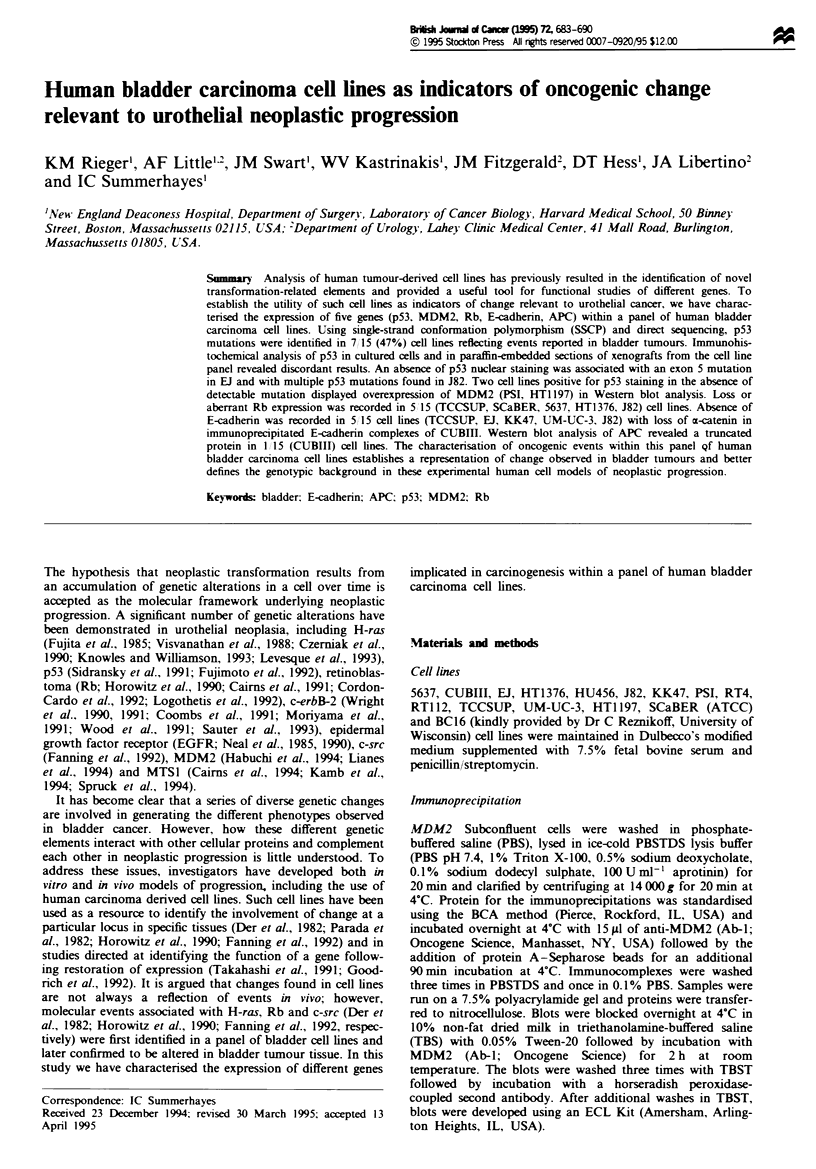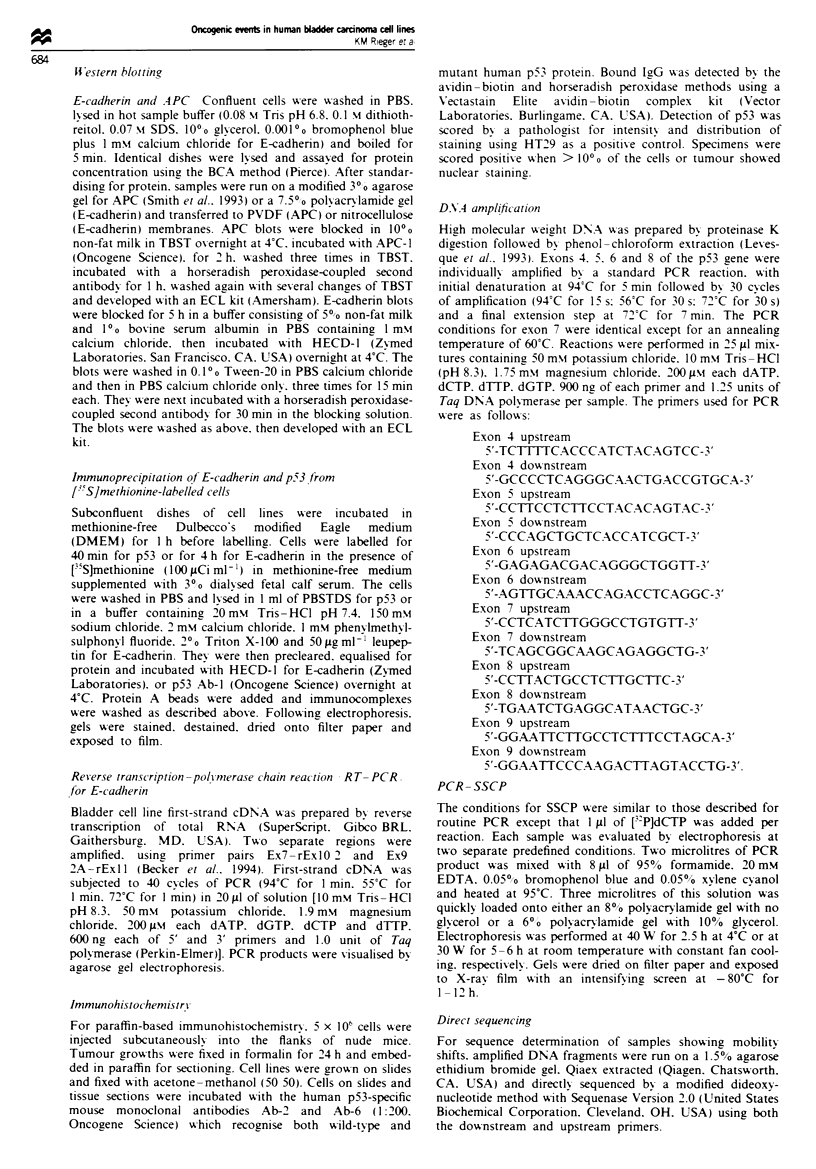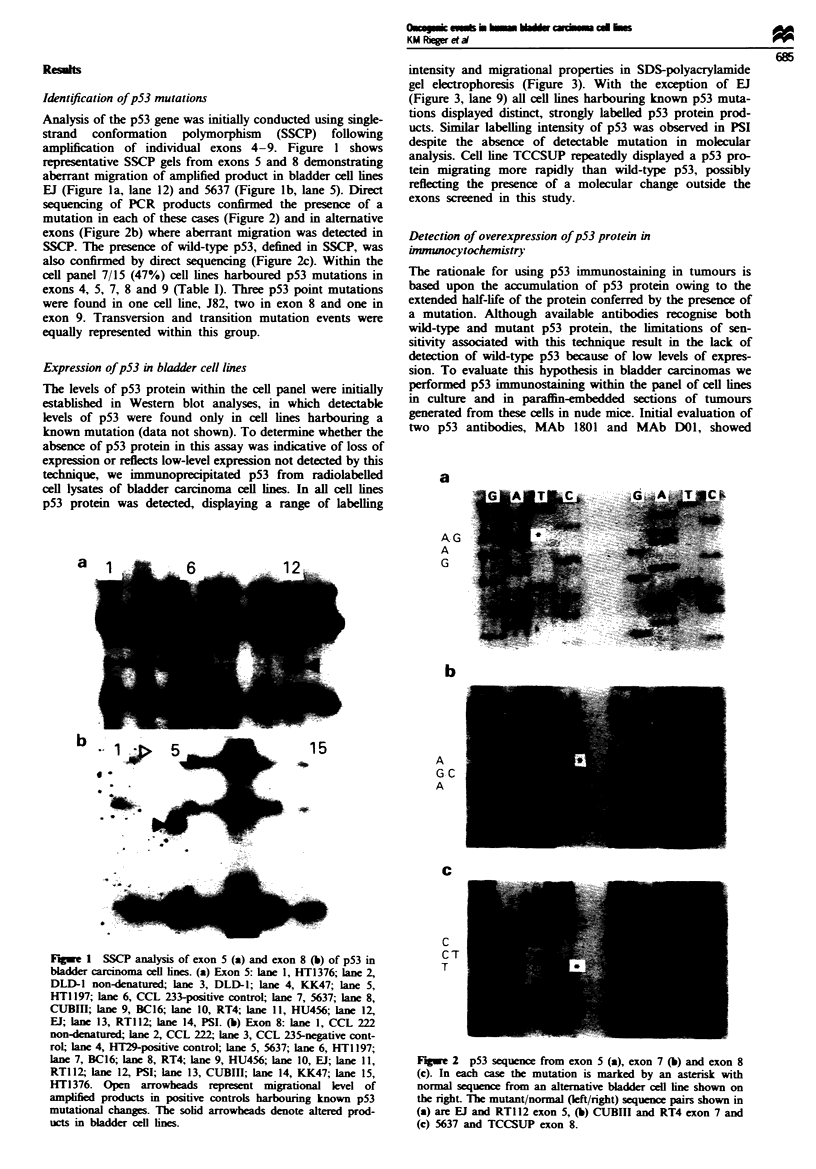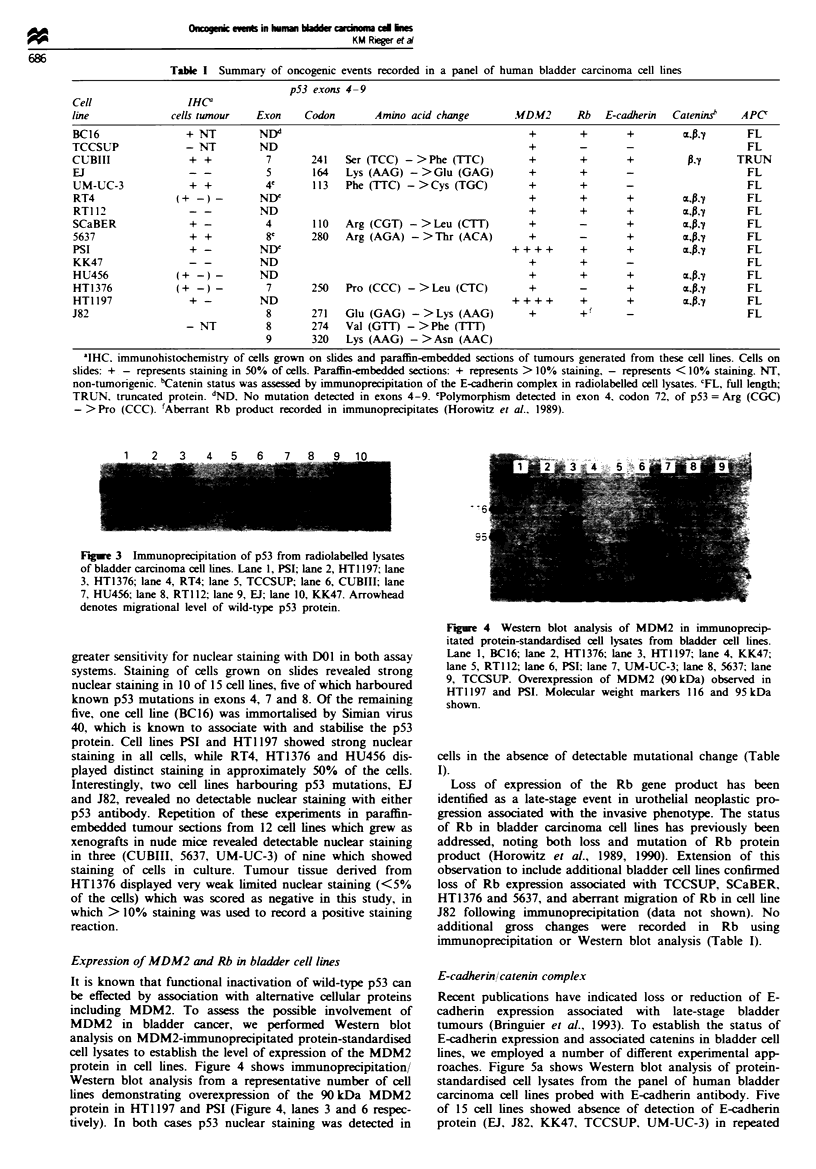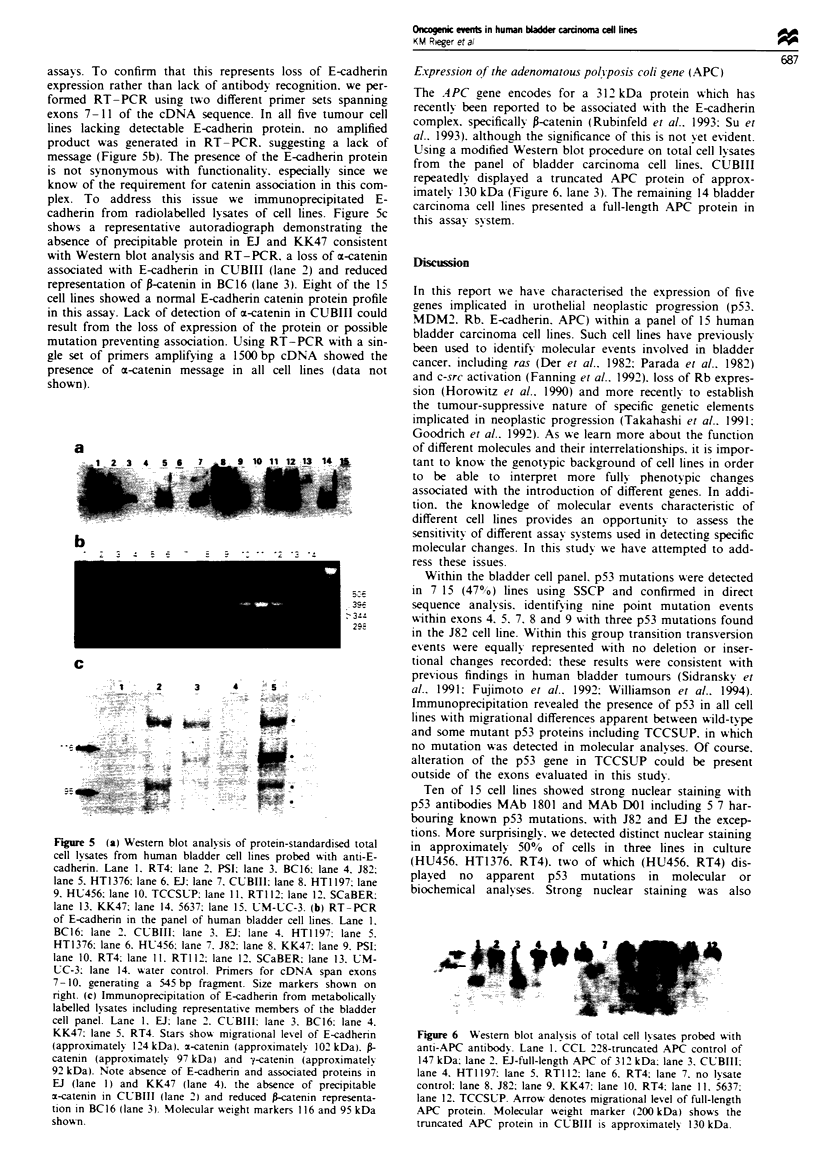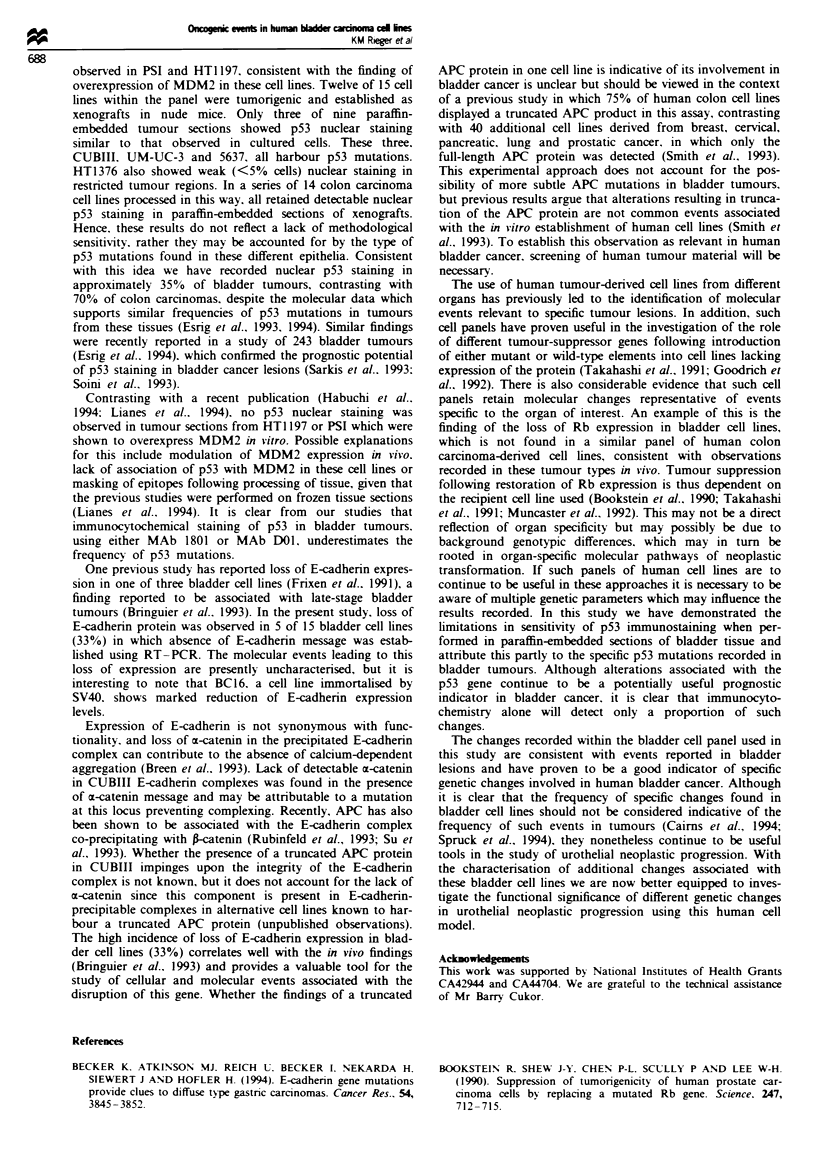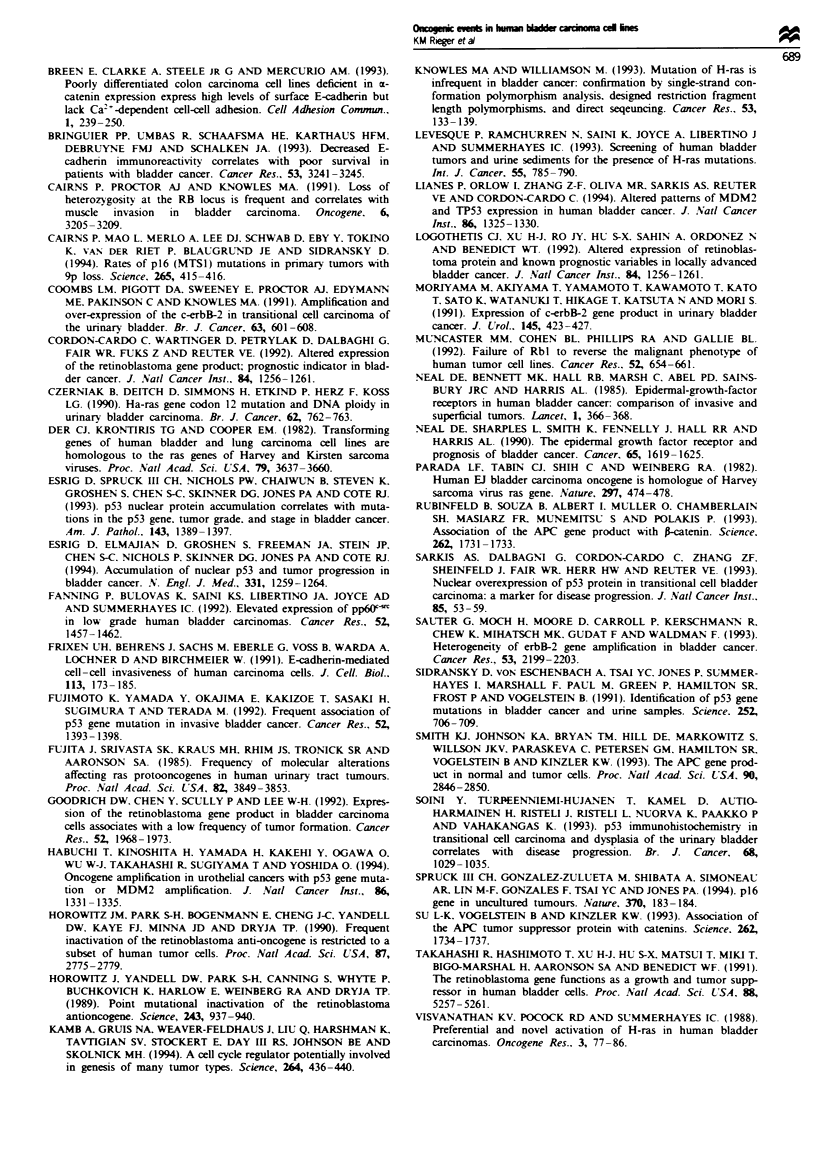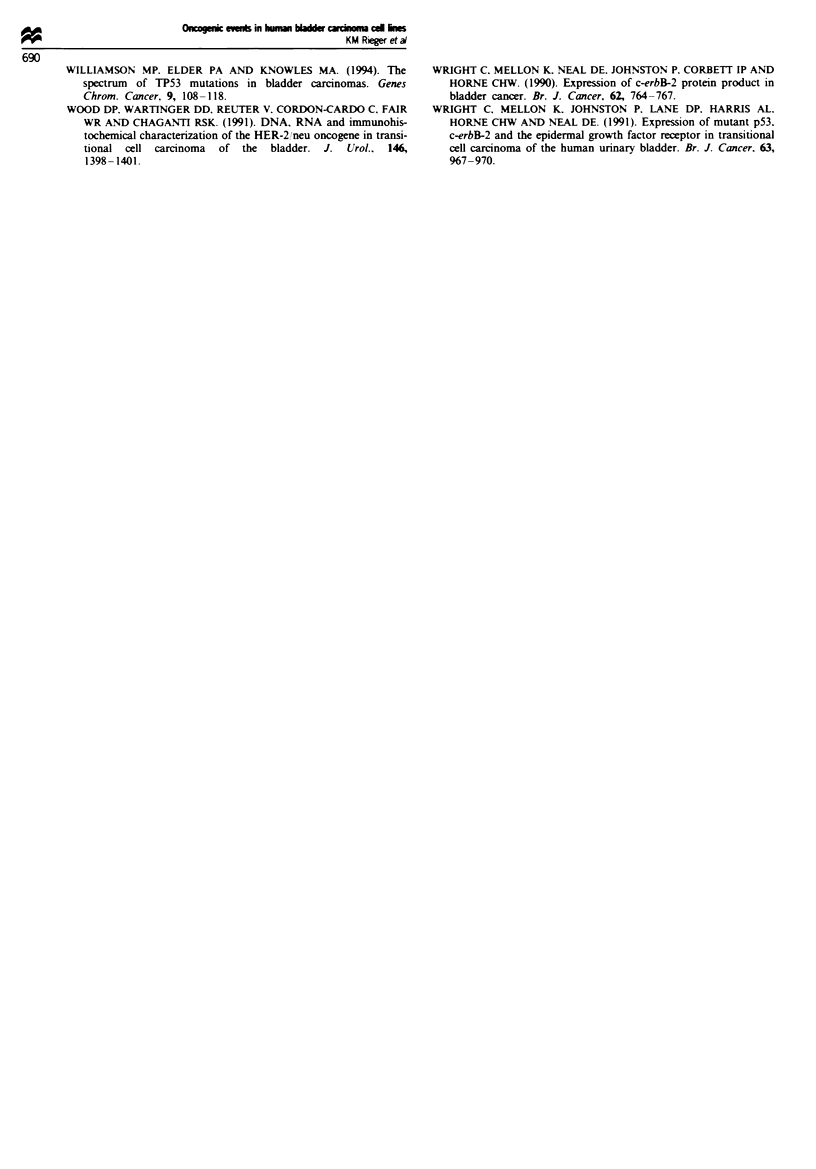# Human bladder carcinoma cell lines as indicators of oncogenic change relevant to urothelial neoplastic progression.

**DOI:** 10.1038/bjc.1995.394

**Published:** 1995-09

**Authors:** K. M. Rieger, A. F. Little, J. M. Swart, W. V. Kastrinakis, J. M. Fitzgerald, D. T. Hess, J. A. Libertino, I. C. Summerhayes

**Affiliations:** New England Deaconess Hospital, Department of Surgery, Harvard Medical School, Boston, Massachussetts 02115, USA.

## Abstract

**Images:**


British Journal of Cancer (1995) 72, 683-690

© 1995 Stockton Press All rights reserved 0007-0920/95 $12.00

### Human bladder carcinoma cell lines as indicators of oncogenic change relevant to urothelial neoplastic progression

KM Rieger[1], AF Little[1,2], JM Swart[1], WV Kastrinakis[1], JM Fitzgerald[2], DT Hess[1], JA Libertino[2] and IC Summerhayes[1]

[1]*New England Deaconess Hospital, Department of Surgery, Laboratory of Cancer Biology, Harvard Medical School, 50 Binney Street, Boston, Massachussetts 02115, USA;* [2]*Department of Urology, Lahey Clinic Medical Center, 41 Mall Road, Burlington, Massachussetts 01805, USA.*

**Summary** Analysis of human tumour-derived cell lines has previously resulted in the identification of novel transformation-related elements and provided a useful tool for functional studies of different genes. To establish the utility of such cell lines as indicators of change relevant to urothelial cancer, we have characterised the expression of five genes (p53, MDM2, Rb, E-cadherin, APC) within a panel of human bladder carcinoma cell lines. Using single-strand conformation polymorphism (SSCP) and direct sequencing, p53 mutations were identified in 7/15 (47%) cell lines reflecting events reported in bladder tumours. Immunohistochemical analysis of p53 in cultured cells and in paraffin-embedded sections of xenografts from the cell line panel revealed discordant results. An absence of p53 nuclear staining was associated with an exon 5 mutation in EJ and with multiple p53 mutations found in J82. Two cell lines positive for p53 staining in the absence of detectable mutation displayed overexpression of MDM2 (PSI, HT1197) in Western blot analysis. Loss or aberrant Rb expression was recorded in 5/15 (TCCSUP, SCaBER, 5637, HT1376, J82) cell lines. Absence of E-cadherin was recorded in 5/15 cell lines (TCCSUP, EJ, KK47, UM-UC-3, J82) with loss of α-catenin in immunoprecipitated E-cadherin complexes of CUBIII. Western blot analysis of APC revealed a truncated protein in 1/15 (CUBIII) cell lines. The characterisation of oncogenic events within this panel of human bladder carcinoma cell lines establishes a representation of change observed in bladder tumours and better defines the genotypic background in these experimental human cell models of neoplastic progression.

**Keywords:** bladder; E-cadherin; APC; p53; MDM2; Rb

The hypothesis that neoplastic transformation results from an accumulation of genetic alterations in a cell over time is accepted as the molecular framework underlying neoplastic progression. A significant number of genetic alterations have been demonstrated in urothelial neoplasia, including H-*ras* (Fujita *et al*., 1985; Visvanathan *et al*., 1988; Czerniak *et al*., 1990; Knowles and Williamson, 1993; Levesque *et al*., 1993), p53 (Sidransky *et al*., 1991; Fujimoto *et al*., 1992), retinoblastoma (Rb; Horowitz *et al*., 1990; Cairns *et al*., 1991; Cordon-Cardo *et al*., 1992; Logothetis *et al*., 1992), c-*erb*B-2 (Wright *et al*., 1990, 1991; Coombs *et al*., 1991; Moriyama *et al*., 1991; Wood *et al*., 1991; Sauter *et al*., 1993), epidermal growth factor receptor (EGFR; Neal *et al*., 1985, 1990), c-*src* (Fanning *et al*., 1992), MDM2 (Habuchi *et al*., 1994; Lianes *et al*., 1994) and MTS1 (Cairns *et al*., 1994; Kamb *et al*., 1994; Spruck *et al*., 1994).

It has become clear that a series of diverse genetic changes are involved in generating the different phenotypes observed in bladder cancer. However, how these different genetic elements interact with other cellular proteins and complement each other in neoplastic progression is little understood. To address these issues, investigators have developed both *in vitro* and *in vivo* models of progression, including the use of human carcinoma derived cell lines. Such cell lines have been used as a resource to identify the involvement of change at a particular locus in specific tissues (Der *et al*., 1982; Parada *et al*., 1982; Horowitz *et al*., 1990; Fanning *et al*., 1992) and in studies directed at identifying the function of a gene following restoration of expression (Takahashi *et al*., 1991; Goodrich *et al*., 1992). It is argued that changes found in cell lines are not always a reflection of events *in vivo*; however, molecular events associated with H-*ras*, Rb and c-*src* (Der *et al*., 1982; Horowitz *et al*., 1990; Fanning *et al*., 1992, respectively) were first identified in a panel of bladder cell lines and later confirmed to be altered in bladder tumour tissue. In this study we have characterised the expression of different genes implicated in carcinogenesis within a panel of human bladder carcinoma cell lines.

Correspondence: IC Summerhayes
Received 23 December 1994; revised 30 March 1995; accepted 13 April 1995

### Materials and methods

### *Cell lines*

5637, CUBIII, EJ, HT1376, HU456, J82, KK47, PSI, RT4, RT112, TCCSUP, UM-UC-3, HT1197, SCaBER (ATCC) and BC16 (kindly provided by Dr C Reznikoff, University of Wisconsin) cell lines were maintained in Dulbecco's modified medium supplemented with 7.5% fetal bovine serum and penicillin/streptomycin.

### *Immunoprecipitation*

*MDM2* Subconfluent cells were washed in phosphate-buffered saline (PBS), lysed in ice-cold PBSTDS lysis buffer (PBS pH 7.4, 1% Triton X-100, 0.5% sodium deoxycholate, 0.1% sodium dodecyl sulphate, 100 U $ml^{-1}$ aprotinin) for 20 min and clarified by centrifuging at 14 000 *g* for 20 min at 4°C. Protein for the immunoprecipitations was standardised using the BCA method (Pierce, Rockford, IL, USA) and incubated overnight at 4°C with 15 μl of anti-MDM2 (Ab-1; Oncogene Science, Manhasset, NY, USA) followed by the addition of protein A-Sepharose beads for an additional 90 min incubation at 4°C. Immunocomplexes were washed three times in PBSTDS and once in 0.1% PBS. Samples were run on a 7.5% polyacrylamide gel and proteins were transferred to nitrocellulose. Blots were blocked overnight at 4°C in 10% non-fat dried milk in triethanolamine-buffered saline (TBS) with 0.05% Tween-20 followed by incubation with MDM2 (Ab-1; Oncogene Science) for 2 h at room temperature. The blots were washed three times with TBST followed by incubation with a horseradish peroxidase-coupled second antibody. After additional washes in TBST, blots were developed using an ECL Kit (Amersham, Arlington Heights, IL, USA).

*Western blotting*

*E-cadherin and APC* Confluent cells were washed in PBS, lysed in hot sample buffer (0.08 M Tris pH 6.8, 0.1 M dithiothreitol, 0.07 M SDS, 10% glycerol, 0.001% bromophenol blue plus 1 mM calcium chloride for E-cadherin) and boiled for 5 min. Identical dishes were lysed and assayed for protein concentration using the BCA method (Pierce). After standardising for protein, samples were run on a modified 3% agarose gel for APC (Smith *et al.*, 1993) or a 7.5% polyacrylamide gel (E-cadherin) and transferred to PVDF (APC) or nitrocellulose (E-cadherin) membranes. APC blots were blocked in 10% non-fat milk in TBST overnight at 4°C, incubated with APC-1 (Oncogene Science), for 2 h, washed three times in TBST, incubated with a horseradish peroxidase-coupled second antibody for 1 h, washed again with several changes of TBST and developed with an ECL kit (Amersham). E-cadherin blots were blocked for 5 h in a buffer consisting of 5% non-fat milk and 1% bovine serum albumin in PBS containing 1 mM calcium chloride, then incubated with HECD-1 (Zymed Laboratories, San Francisco, CA, USA) overnight at 4°C. The blots were washed in 0.1% Tween-20 in PBS calcium chloride and then in PBS calcium chloride only, three times for 15 min each. They were next incubated with a horseradish peroxidase-coupled second antibody for 30 min in the blocking solution. The blots were washed as above, then developed with an ECL kit.

*Immunoprecipitation of E-cadherin and p53 from [$^{35}$S]methionine-labelled cells*

Subconfluent dishes of cell lines were incubated in methionine-free Dulbecco's modified Eagle medium (DMEM) for 1 h before labelling. Cells were labelled for 40 min for p53 or for 4 h for E-cadherin in the presence of [$^{35}$S]methionine (100 μCi ml$^{-1}$) in methionine-free medium supplemented with 3% dialysed fetal calf serum. The cells were washed in PBS and lysed in 1 ml of PBSTDS for p53 or in a buffer containing 20 mM Tris-HCl pH 7.4, 150 mM sodium chloride, 2 mM calcium chloride, 1 mM phenylmethylsulphonyl fluoride, 2% Triton X-100 and 50 μg ml$^{-1}$ leupeptin for E-cadherin. They were then precleared, equalised for protein and incubated with HECD-1 for E-cadherin (Zymed Laboratories), or p53 Ab-1 (Oncogene Science) overnight at 4°C. Protein A beads were added and immunocomplexes were washed as described above. Following electrophoresis, gels were stained, destained, dried onto filter paper and exposed to film.

*Reverse transcription-polymerase chain reaction (RT-PCR) for E-cadherin*

Bladder cell line first-strand cDNA was prepared by reverse transcription of total RNA (SuperScript, Gibco BRL, Gaithersburg, MD, USA). Two separate regions were amplified, using primer pairs Ex7-rEx10/2 and Ex9/2A-rEx11 (Becker *et al.*, 1994). First-strand cDNA was subjected to 40 cycles of PCR (94°C for 1 min, 55°C for 1 min, 72°C for 1 min) in 20 μl of solution [10 mM Tris-HCl pH 8.3, 50 mM potassium chloride, 1.9 mM magnesium chloride, 200 μM each dATP, dGTP, dCTP and dTTP, 600 ng each of 5′ and 3′ primers and 1.0 unit of *Taq* polymerase (Perkin-Elmer)]. PCR products were visualised by agarose gel electrophoresis.

*Immunohistochemistry*

For paraffin-based immunohistochemistry, 5 × 10$^6$ cells were injected subcutaneously into the flanks of nude mice. Tumour growths were fixed in formalin for 24 h and embedded in paraffin for sectioning. Cell lines were grown on slides and fixed with acetone-methanol (50/50). Cells on slides and tissue sections were incubated with the human p53-specific mouse monoclonal antibodies Ab-2 and Ab-6 (1:200, Oncogene Science) which recognise both wild-type and mutant human p53 protein. Bound IgG was detected by the avidin-biotin and horseradish peroxidase methods using a Vectastain Elite avidin-biotin complex kit (Vector Laboratories, Burlingame, CA, USA). Detection of p53 was scored by a pathologist for intensity and distribution of staining using HT29 as a positive control. Specimens were scored positive when > 10% of the cells or tumour showed nuclear staining.

*DNA amplification*

High molecular weight DNA was prepared by proteinase K digestion followed by phenol-chloroform extraction (Levesque *et al.*, 1993). Exons 4, 5, 6 and 8 of the p53 gene were individually amplified by a standard PCR reaction, with initial denaturation at 94°C for 5 min followed by 30 cycles of amplification (94°C for 15 s; 56°C for 30 s; 72°C for 30 s) and a final extension step at 72°C for 7 min. The PCR conditions for exon 7 were identical except for an annealing temperature of 60°C. Reactions were performed in 25 μl mixtures containing 50 mM potassium chloride, 10 mM Tris-HCl (pH 8.3), 1.75 mM magnesium chloride, 200 μM each dATP, dCTP, dTTP, dGTP, 900 ng of each primer and 1.25 units of *Taq* DNA polymerase per sample. The primers used for PCR were as follows:

Exon 4 upstream
5′-TCTTTTCACCCATCTACAGTCC-3′
Exon 4 downstream
5′-GCCCCTCAGGGCAACTGACCGTGCA-3′
Exon 5 upstream
5′-CCTTCCTCTTCCTACACAGTAC-3′
Exon 5 downstream
5′-CCCAGCTGCTCACCATCGCT-3′
Exon 6 upstream
5′-GAGAGACGACAGGGCTGGTT-3′
Exon 6 downstream
5′-AGTTGCAAACCAGACCTCAGGC-3′
Exon 7 upstream
5′-CCTCATCTTGGGCCTGTGTT-3′
Exon 7 downstream
5′-TCAGCGGCAAGCAGAGGCTG-3′
Exon 8 upstream
5′-CCTTACTGCCTCTTGCTTC-3′
Exon 8 downstream
5′-TGAATCTGAGGCATAACTGC-3′
Exon 9 upstream
5′-GGAATTCTTGCCTCTTTCCTAGCA-3′
Exon 9 downstream
5′-GGAATTCCCAAGACTTAGTACCTG-3′.

*PCR-SSCP*

The conditions for SSCP were similar to those described for routine PCR except that 1 μl of [$^{32}$P]dCTP was added per reaction. Each sample was evaluated by electrophoresis at two separate predefined conditions. Two microlitres of PCR product was mixed with 8 μl of 95% formamide, 20 mM EDTA, 0.05% bromophenol blue and 0.05% xylene cyanol and heated at 95°C. Three microlitres of this solution was quickly loaded onto either an 8% polyacrylamide gel with no glycerol or a 6% polyacrylamide gel with 10% glycerol. Electrophoresis was performed at 40 W for 2.5 h at 4°C or at 30 W for 5-6 h at room temperature with constant fan cooling, respectively. Gels were dried on filter paper and exposed to X-ray film with an intensifying screen at −80°C for 1-12 h.

*Direct sequencing*

For sequence determination of samples showing mobility shifts, amplified DNA fragments were run on a 1.5% agarose ethidium bromide gel, Qiaex extracted (Qiagen, Chatsworth, CA, USA) and directly sequenced by a modified dideoxynucleotide method with Sequenase Version 2.0 (United States Biochemical Corporation, Cleveland, OH, USA) using both the downstream and upstream primers.

### Results

### *Identification of p53 mutations*

Analysis of the p53 gene was initially conducted using single-strand conformation polymorphism (SSCP) following amplification of individual exons 4–9. Figure 1 shows representative SSCP gels from exons 5 and 8 demonstrating aberrant migration of amplified product in bladder cell lines EJ (Figure 1a, lane 12) and 5637 (Figure 1b, lane 5). Direct sequencing of PCR products confirmed the presence of a mutation in each of these cases (Figure 2) and in alternative exons (Figure 2b) where aberrant migration was detected in SSCP. The presence of wild-type p53, defined in SSCP, was also confirmed by direct sequencing (Figure 2c). Within the cell panel 7/15 (47%) cell lines harboured p53 mutations in exons 4, 5, 7, 8 and 9 (Table I). Three p53 point mutations were found in one cell line, J82, two in exon 8 and one in exon 9. Transversion and transition mutation events were equally represented within this group.

### *Expression of p53 in bladder cell lines*

The levels of p53 protein within the cell panel were initially established in Western blot analyses, in which detectable levels of p53 were found only in cell lines harbouring a known mutation (data not shown). To determine whether the absence of p53 protein in this assay was indicative of loss of expression or reflects low-level expression not detected by this technique, we immunoprecipitated p53 from radiolabelled cell lysates of bladder carcinoma cell lines. In all cell lines p53 protein was detected, displaying a range of labelling intensity and migrational properties in SDS-polyacrylamide gel electrophoresis (Figure 3). With the exception of EJ (Figure 3, lane 9) all cell lines harbouring known p53 mutations displayed distinct, strongly labelled p53 protein products. Similar labelling intensity of p53 was observed in PSI despite the absence of detectable mutation in molecular analysis. Cell line TCCSUP repeatedly displayed a p53 protein migrating more rapidly than wild-type p53, possibly reflecting the presence of a molecular change outside the exons screened in this study.

### *Detection of overexpression of p53 protein in immunocytochemistry*

The rationale for using p53 immunostaining in tumours is based upon the accumulation of p53 protein owing to the extended half-life of the protein conferred by the presence of a mutation. Although available antibodies recognise both wild-type and mutant p53 protein, the limitations of sensitivity associated with this technique result in the lack of detection of wild-type p53 because of low levels of expression. To evaluate this hypothesis in bladder carcinomas we performed p53 immunostaining within the panel of cell lines in culture and in paraffin-embedded sections of tumours generated from these cells in nude mice. Initial evaluation of two p53 antibodies, MAb 1801 and MAb D01, showed

**Figure 1** SSCP analysis of exon 5 (**a**) and exon 8 (**b**) of p53 in bladder carcinoma cell lines. (**a**) Exon 5: lane 1, HT1376; lane 2, DLD-1 non-denatured; lane 3, DLD-1; lane 4, KK47; lane 5, HT1197; lane 6, CCL 233-positive control; lane 7, 5637; lane 8, CUBIII; lane 9, BC16; lane 10, RT4; lane 11, HU456; lane 12, EJ; lane 13, RT112; lane 14, PSI. (**b**) Exon 8: lane 1, CCL 222 non-denatured; lane 2, CCL 222; lane 3, CCL 235-negative control; lane 4, HT29-positive control; lane 5, 5637; lane 6, HT1197; lane 7, BC16; lane 8, RT4; lane 9, HU456; lane 10, EJ; lane 11, RT112; lane 12, PSI; lane 13, CUBIII; lane 14, KK47; lane 15, HT1376. Open arrowheads represent migrational level of amplified products in positive controls harbouring known p53 mutational changes. The solid arrowheads denote altered products in bladder cell lines.

**Figure 2** p53 sequence from exon 5 (**a**), exon 7 (**b**) and exon 8 (**c**). In each case the mutation is marked by an asterisk with normal sequence from an alternative bladder cell line shown on the right. The mutant/normal (left/right) sequence pairs shown in (**a**) are EJ and RT112 exon 5, (**b**) CUBIII and RT4 exon 7 and (**c**) 5637 and TCCSUP exon 8.

**Table I** Summary of oncogenic events recorded in a panel of human bladder carcinoma cell lines

| Cell line | IHC[a] cells tumour | p53 exons 4–9 Exon | Codon | Amino acid change | MDM2 | Rb | E-cadherin | Catenins[b] | APC[c] |
|---|---|---|---|---|---|---|---|---|---|
| BC16 | + NT | ND[d] | | | + | + | + | $\alpha,\beta,\gamma$ | FL |
| TCCSUP | – NT | ND | | | + | – | – | | FL |
| CUBIII | + + | 7 | 241 | Ser (TCC) – > Phe (TTC) | + | + | + | $\beta,\gamma$ | TRUN |
| EJ | – – | 5 | 164 | Lys (AAG) – > Glu (GAG) | + | + | – | | FL |
| UM-UC-3 | + + | 4[e] | 113 | Phe (TTC) – > Cys (TGC) | + | + | – | | FL |
| RT4 | (+ –) – | ND[e] | | | + | + | + | $\alpha,\beta,\gamma$ | FL |
| RT112 | – – | ND | | | + | + | + | $\alpha,\beta,\gamma$ | FL |
| SCaBER | + – | 4 | 110 | Arg (CGT) – > Leu (CTT) | + | – | + | $\alpha,\beta,\gamma$ | FL |
| 5637 | + + | 8[e] | 280 | Arg (AGA) – > Thr (ACA) | + | – | + | $\alpha,\beta,\gamma$ | FL |
| PSI | + – | ND[e] | | | + + + + | + | + | $\alpha,\beta,\gamma$ | FL |
| KK47 | – – | ND | | | + | + | – | | FL |
| HU456 | (+ –) – | ND | | | + | + | + | $\alpha,\beta,\gamma$ | FL |
| HT1376 | (+ –) – | 7 | 250 | Pro (CCC) – > Leu (CTC) | + | – | + | $\alpha,\beta,\gamma$ | FL |
| HT1197 | + – | ND | | | + + + + | + | + | $\alpha,\beta,\gamma$ | FL |
| J82 | | 8 | 271 | Glu (GAG) – > Lys (AAG) | + | +[f] | – | | FL |
| | – NT | 8 | 274 | Val (GTT) – > Phe (TTT) | | | | | |
| | | 9 | 320 | Lys (AAG) – > Asn (AAC) | | | | | |

[a]IHC, immunohistochemistry of cells grown on slides and paraffin-embedded sections of tumours generated from these cell lines. Cells on slides: + – represents staining in 50% of cells. Paraffin-embedded sections: + represents > 10% staining, – represents < 10% staining. NT, non-tumorigenic. [b]Catenin status was assessed by immunoprecipitation of the E-cadherin complex in radiolabelled cell lysates. [c]FL, full length; TRUN, truncated protein. [d]ND, No mutation detected in exons 4–9. [e]Polymorphism detected in exon 4, codon 72, of p53 = Arg (CGC) – > Pro (CCC). [f]Aberrant Rb product recorded in immunoprecipitates (Horowitz *et al*., 1989).

**Figure 3** Immunoprecipitation of p53 from radiolabelled lysates of bladder carcinoma cell lines. Lane 1, PSI; lane 2, HT1197; lane 3, HT1376; lane 4, RT4; lane 5, TCCSUP; lane 6, CUBIII; lane 7, HU456; lane 8, RT112; lane 9, EJ; lane 10, KK47. Arrowhead denotes migrational level of wild-type p53 protein.

**Figure 4** Western blot analysis of MDM2 in immunoprecipitated protein-standardised cell lysates from bladder cell lines. Lane 1, BC16; lane 2, HT1376; lane 3, HT1197; lane 4, KK47; lane 5, RT112; lane 6, PSI; lane 7, UM-UC-3; lane 8, 5637; lane 9, TCCSUP. Overexpression of MDM2 (90 kDa) observed in HT1197 and PSI. Molecular weight markers 116 and 95 kDa shown.

greater sensitivity for nuclear staining with DO1 in both assay systems. Staining of cells grown on slides revealed strong nuclear staining in 10 of 15 cell lines, five of which harboured known p53 mutations in exons 4, 7 and 8. Of the remaining five, one cell line (BC16) was immortalised by Simian virus 40, which is known to associate with and stabilise the p53 protein. Cell lines PSI and HT1197 showed strong nuclear staining in all cells, while RT4, HT1376 and HU456 displayed distinct staining in approximately 50% of the cells. Interestingly, two cell lines harbouring p53 mutations, EJ and J82, revealed no detectable nuclear staining with either p53 antibody. Repetition of these experiments in paraffin-embedded tumour sections from 12 cell lines which grew as xenografts in nude mice revealed detectable nuclear staining in three (CUBIII, 5637, UM-UC-3) of nine which showed staining of cells in culture. Tumour tissue derived from HT1376 displayed very weak limited nuclear staining (<5% of the cells) which was scored as negative in this study, in which > 10% staining was used to record a positive staining reaction.

*Expression of MDM2 and Rb in bladder cell lines*

It is known that functional inactivation of wild-type p53 can be effected by association with alternative cellular proteins including MDM2. To assess the possible involvement of MDM2 in bladder cancer, we performed Western blot analysis on MDM2-immunoprecipitated protein-standardised cell lysates to establish the level of expression of the MDM2 protein in cell lines. Figure 4 shows immunoprecipitation/Western blot analysis from a representative number of cell lines demonstrating overexpression of the 90 kDa MDM2 protein in HT1197 and PSI (Figure 4, lanes 3 and 6 respectively). In both cases p53 nuclear staining was detected in cells in the absence of detectable mutational change (Table I).

Loss of expression of the Rb gene product has been identified as a late-stage event in urothelial neoplastic progression associated with the invasive phenotype. The status of Rb in bladder carcinoma cell lines has previously been addressed, noting both loss and mutation of Rb protein product (Horowitz *et al*., 1989, 1990). Extension of this observation to include additional bladder cell lines confirmed loss of Rb expression associated with TCCSUP, SCaBER, HT1376 and 5637, and aberrant migration of Rb in cell line J82 following immunoprecipitation (data not shown). No additional gross changes were recorded in Rb using immunoprecipitation or Western blot analysis (Table I).

*E-cadherin/catenin complex*

Recent publications have indicated loss or reduction of E-cadherin expression associated with late-stage bladder tumours (Bringuier *et al*., 1993). To establish the status of E-cadherin expression and associated catenins in bladder cell lines, we employed a number of different experimental approaches. Figure 5a shows Western blot analysis of protein-standardised cell lysates from the panel of human bladder carcinoma cell lines probed with E-cadherin antibody. Five of 15 cell lines showed absence of detection of E-cadherin protein (EJ, J82, KK47, TCCSUP, UM-UC-3) in repeated assays. To confirm that this represents loss of E-cadherin expression rather than lack of antibody recognition, we performed RT–PCR using two different primer sets spanning exons 7–11 of the cDNA sequence. In all five tumour cell lines lacking detectable E-cadherin protein, no amplified product was generated in RT–PCR, suggesting a lack of message (Figure 5b). The presence of the E-cadherin protein is not synonymous with functionality, especially since we know of the requirement for catenin association in this complex. To address this issue we immunoprecipitated E-cadherin from radiolabelled lysates of cell lines. Figure 5c shows a representative autoradiograph demonstrating the absence of precipitable protein in EJ and KK47 consistent with Western blot analysis and RT–PCR, a loss of α-catenin associated with E-cadherin in CUBIII (lane 2) and reduced representation of β-catenin in BC16 (lane 3). Eight of the 15 cell lines showed a normal E-cadherin catenin protein profile in this assay. Lack of detection of α-catenin in CUBIII could result from the loss of expression of the protein or possible mutation preventing association. Using RT–PCR with a single set of primers amplifying a 1500 bp cDNA showed the presence of α-catenin message in all cell lines (data not shown).

**Figure 5** (**a**) Western blot analysis of protein-standardised total cell lysates from human bladder cell lines probed with anti-E-cadherin. Lane 1, RT4; lane 2, PSI; lane 3, BC16; lane 4, J82; lane 5, HT1376; lane 6, EJ; lane 7, CUBIII; lane 8, HT1197; lane 9, HU456; lane 10, TCCSUP; lane 11, RT112; lane 12, SCaBER; lane 13, KK47; lane 14, 5637; lane 15, UM-UC-3. (**b**) RT–PCR of E-cadherin in the panel of human bladder cell lines. Lane 1, BC16; lane 2, CUBIII; lane 3, EJ; lane 4, HT1197; lane 5, HT1376; lane 6, HU456; lane 7, J82; lane 8, KK47; lane 9, PSI; lane 10, RT4; lane 11, RT112; lane 12, SCaBER; lane 13, UM-UC-3; lane 14, water control. Primers for cDNA span exons 7–10, generating a 545 bp fragment. Size markers shown on right. (**c**) Immunoprecipitation of E-cadherin from metabolically labelled lysates including representative members of the bladder cell panel. Lane 1, EJ; lane 2, CUBIII; lane 3, BC16; lane 4, KK47; lane 5, RT4. Stars show migrational level of E-cadherin (approximately 124 kDa), α-catenin (approximately 102 kDa), β-catenin (approximately 97 kDa) and γ-catenin (approximately 92 kDa). Note absence of E-cadherin and associated proteins in EJ (lane 1) and KK47 (lane 4), the absence of precipitable α-catenin in CUBIII (lane 2) and reduced β-catenin representation in BC16 (lane 3). Molecular weight markers 116 and 95 kDa shown.

*Expression of the adenomatous polyposis coli gene* (APC)

The *APC* gene encodes for a 312 kDa protein which has recently been reported to be associated with the E-cadherin complex, specifically β-catenin (Rubinfeld *et al*., 1993; Su *et al*., 1993), although the significance of this is not yet evident. Using a modified Western blot procedure on total cell lysates from the panel of bladder carcinoma cell lines, CUBIII repeatedly displayed a truncated APC protein of approximately 130 kDa (Figure 6, lane 3). The remaining 14 bladder carcinoma cell lines presented a full-length APC protein in this assay system.

### Discussion

In this report we have characterised the expression of five genes implicated in urothelial neoplastic progression (p53, MDM2, Rb, E-cadherin, APC) within a panel of 15 human bladder carcinoma cell lines. Such cell lines have previously been used to identify molecular events involved in bladder cancer, including *ras* (Der *et al*., 1982; Parada *et al*., 1982) and c-*src* activation (Fanning *et al*., 1992), loss of Rb expression (Horowitz *et al*., 1990) and more recently to establish the tumour-suppressive nature of specific genetic elements implicated in neoplastic progression (Takahashi *et al*., 1991; Goodrich *et al*., 1992). As we learn more about the function of different molecules and their interrelationships, it is important to know the genotypic background of cell lines in order to be able to interpret more fully phenotypic changes associated with the introduction of different genes. In addition, the knowledge of molecular events characteristic of different cell lines provides an opportunity to assess the sensitivity of different assay systems used in detecting specific molecular changes. In this study we have attempted to address these issues.

Within the bladder cell panel, p53 mutations were detected in 7 15 (47%) lines using SSCP and confirmed in direct sequence analysis, identifying nine point mutation events within exons 4, 5, 7, 8 and 9 with three p53 mutations found in the J82 cell line. Within this group transition transversion events were equally represented with no deletion or insertional changes recorded; these results were consistent with previous findings in human bladder tumours (Sidransky *et al*., 1991; Fujimoto *et al*., 1992; Williamson *et al*., 1994). Immunoprecipitation revealed the presence of p53 in all cell lines with migrational differences apparent between wild-type and some mutant p53 proteins including TCCSUP, in which no mutation was detected in molecular analyses. Of course, alteration of the p53 gene in TCCSUP could be present outside of the exons evaluated in this study.

Ten of 15 cell lines showed strong nuclear staining with p53 antibodies MAb 1801 and MAb D01 including 5 7 harbouring known p53 mutations, with J82 and EJ the exceptions. More surprisingly, we detected distinct nuclear staining in approximately 50% of cells in three lines in culture (HU456, HT1376, RT4), two of which (HU456, RT4) displayed no apparent p53 mutations in molecular or biochemical analyses. Strong nuclear staining was also

**Figure 6** Western blot analysis of total cell lysates probed with anti-APC antibody. Lane 1, CCL 228-truncated APC control of 147 kDa; lane 2, EJ-full-length APC of 312 kDa; lane 3, CUBIII; lane 4, HT1197; lane 5, RT112; lane 6, RT4; lane 7, no lysate control; lane 8, J82; lane 9, KK47; lane 10, RT4; lane 11, 5637; lane 12, TCCSUP. Arrow denotes migrational level of full-length APC protein. Molecular weight marker (200 kDa) shows the truncated APC protein in CUBIII is approximately 130 kDa.

observed in PSI and HT1197, consistent with the finding of overexpression of MDM2 in these cell lines. Twelve of 15 cell lines within the panel were tumorigenic and established as xenografts in nude mice. Only three of nine paraffin-embedded tumour sections showed p53 nuclear staining similar to that observed in cultured cells. These three, CUBIII, UM-UC-3 and 5637, all harbour p53 mutations. HT1376 also showed weak (<5% cells) nuclear staining in restricted tumour regions. In a series of 14 colon carcinoma cell lines processed in this way, all retained detectable nuclear p53 staining in paraffin-embedded sections of xenografts. Hence, these results do not reflect a lack of methodological sensitivity, rather they may be accounted for by the type of p53 mutations found in these different epithelia. Consistent with this idea we have recorded nuclear p53 staining in approximately 35% of bladder tumours, contrasting with 70% of colon carcinomas, despite the molecular data which supports similar frequencies of p53 mutations in tumours from these tissues (Esrig *et al.*, 1993, 1994). Similar findings were recently reported in a study of 243 bladder tumours (Esrig *et al.*, 1994), which confirmed the prognostic potential of p53 staining in bladder cancer lesions (Sarkis *et al.*, 1993; Soini *et al.*, 1993).

Contrasting with a recent publication (Habuchi *et al.*, 1994; Lianes *et al.*, 1994), no p53 nuclear staining was observed in tumour sections from HT1197 or PSI which were shown to overexpress MDM2 *in vitro*. Possible explanations for this include modulation of MDM2 expression *in vivo*, lack of association of p53 with MDM2 in these cell lines or masking of epitopes following processing of tissue, given that the previous studies were performed on frozen tissue sections (Lianes *et al.*, 1994). It is clear from our studies that immunocytochemical staining of p53 in bladder tumours, using either MAb 1801 or MAb D01, underestimates the frequency of p53 mutations.

One previous study has reported loss of E-cadherin expression in one of three bladder cell lines (Frixen *et al.*, 1991), a finding reported to be associated with late-stage bladder tumours (Bringuier *et al.*, 1993). In the present study, loss of E-cadherin protein was observed in 5 of 15 bladder cell lines (33%) in which absence of E-cadherin message was established using RT-PCR. The molecular events leading to this loss of expression are presently uncharacterised, but it is interesting to note that BC16, a cell line immortalised by SV40, shows marked reduction of E-cadherin expression levels.

Expression of E-cadherin is not synonymous with functionality, and loss of α-catenin in the precipitated E-cadherin complex can contribute to the absence of calcium-dependent aggregation (Breen *et al.*, 1993). Lack of detectable α-catenin in CUBIII E-cadherin complexes was found in the presence of α-catenin message and may be attributable to a mutation at this locus preventing complexing. Recently, APC has also been shown to be associated with the E-cadherin complex co-precipitating with β-catenin (Rubinfeld *et al.*, 1993; Su *et al.*, 1993). Whether the presence of a truncated APC protein in CUBIII impinges upon the integrity of the E-cadherin complex is not known, but it does not account for the lack of α-catenin since this component is present in E-cadherin-precipitable complexes in alternative cell lines known to harbour a truncated APC protein (unpublished observations). The high incidence of loss of E-cadherin expression in bladder cell lines (33%) correlates well with the *in vivo* findings (Bringuier *et al.*, 1993) and provides a valuable tool for the study of cellular and molecular events associated with the disruption of this gene. Whether the findings of a truncated APC protein in one cell line is indicative of its involvement in bladder cancer is unclear but should be viewed in the context of a previous study in which 75% of human colon cell lines displayed a truncated APC product in this assay, contrasting with 40 additional cell lines derived from breast, cervical, pancreatic, lung and prostatic cancer, in which only the full-length APC protein was detected (Smith *et al.*, 1993). This experimental approach does not account for the possibility of more subtle APC mutations in bladder tumours, but previous results argue that alterations resulting in truncation of the APC protein are not common events associated with the *in vitro* establishment of human cell lines (Smith *et al.*, 1993). To establish this observation as relevant in human bladder cancer, screening of human tumour material will be necessary.

The use of human tumour-derived cell lines from different organs has previously led to the identification of molecular events relevant to specific tumour lesions. In addition, such cell panels have proven useful in the investigation of the role of different tumour-suppressor genes following introduction of either mutant or wild-type elements into cell lines lacking expression of the protein (Takahashi *et al.*, 1991; Goodrich *et al.*, 1992). There is also considerable evidence that such cell panels retain molecular changes representative of events specific to the organ of interest. An example of this is the finding of the loss of Rb expression in bladder cell lines, which is not found in a similar panel of human colon carcinoma-derived cell lines, consistent with observations recorded in these tumour types *in vivo*. Tumour suppression following restoration of Rb expression is thus dependent on the recipient cell line used (Bookstein *et al.*, 1990; Takahashi *et al.*, 1991; Muncaster *et al.*, 1992). This may not be a direct reflection of organ specificity but may possibly be due to background genotypic differences, which may in turn be rooted in organ-specific molecular pathways of neoplastic transformation. If such panels of human cell lines are to continue to be useful in these approaches it is necessary to be aware of multiple genetic parameters which may influence the results recorded. In this study we have demonstrated the limitations in sensitivity of p53 immunostaining when performed in paraffin-embedded sections of bladder tissue and attribute this partly to the specific p53 mutations recorded in bladder tumours. Although alterations associated with the p53 gene continue to be a potentially useful prognostic indicator in bladder cancer, it is clear that immunocytochemistry alone will detect only a proportion of such changes.

The changes recorded within the bladder cell panel used in this study are consistent with events reported in bladder lesions and have proven to be a good indicator of specific genetic changes involved in human bladder cancer. Although it is clear that the frequency of specific changes found in bladder cell lines should not be considered indicative of the frequency of such events in tumours (Cairns *et al.*, 1994; Spruck *et al.*, 1994), they nonetheless continue to be useful tools in the study of urothelial neoplastic progression. With the characterisation of additional changes associated with these bladder cell lines we are now better equipped to investigate the functional significance of different genetic changes in urothelial neoplastic progression using this human cell model.

**Acknowledgements**

This work was supported by National Institutes of Health Grants CA42944 and CA44704. We are grateful to the technical assistance of Mr Barry Cukor.

### References

BECKER K, ATKINSON MJ, REICH U, BECKER I, NEKARDA H, SIEWERT J AND HOFLER H. (1994). E-cadherin gene mutations provide clues to diffuse type gastric carcinomas. *Cancer Res.*, **54**, 3845–3852.

BOOKSTEIN R, SHEW J-Y, CHEN P-L, SCULLY P AND LEE W-H. (1990). Suppression of tumorigenicity of human prostate carcinoma cells by replacing a mutated Rb gene. *Science*, **247**, 712–715.

BREEN E, CLARKE A, STEELE JR G AND MERCURIO AM. (1993). Poorly differentiated colon carcinoma cell lines deficient in α-catenin expression express high levels of surface E-cadherin but lack $Ca^{2+}$-dependent cell-cell adhesion. *Cell Adhesion Commun.*, **1**, 239–250.

BRINGUIER PP, UMBAS R, SCHAAFSMA HE, KARTHAUS HFM, DEBRUYNE FMJ AND SCHALKEN JA. (1993). Decreased E-cadherin immunoreactivity correlates with poor survival in patients with bladder cancer. *Cancer Res.*, **53**, 3241–3245.

CAIRNS P, PROCTOR AJ AND KNOWLES MA. (1991). Loss of heterozygosity at the RB locus is frequent and correlates with muscle invasion in bladder carcinoma. *Oncogene*, **6**, 3205–3209.

CAIRNS P, MAO L, MERLO A, LEE DJ, SCHWAB D, EBY Y, TOKINO K, VAN DER RIET P, BLAUGRUND JE AND SIDRANSKY D. (1994). Rates of p16 (MTS1) mutations in primary tumors with 9p loss. *Science*, **265**, 415–416.

COOMBS LM, PIGOTT DA, SWEENEY E, PROCTOR AJ, EDYMANN ME, PAKINSON C AND KNOWLES MA. (1991). Amplification and over-expression of the c-erbB-2 in transitional cell carcinoma of the urinary bladder. *Br. J. Cancer*, **63**, 601–608.

CORDON-CARDO C, WARTINGER D, PETRYLAK D, DALBAGHI G, FAIR WR, FUKS Z AND REUTER VE. (1992). Altered expression of the retinoblastoma gene product; prognostic indicator in bladder cancer. *J. Natl Cancer Inst.*, **84**, 1256–1261.

CZERNIAK B, DEITCH D, SIMMONS H, ETKIND P, HERZ F, KOSS LG. (1990). Ha-ras gene codon 12 mutation and DNA ploidy in urinary bladder carcinoma. *Br. J. Cancer*, **62**, 762–763.

DER CJ, KRONTIRIS TG AND COOPER EM. (1982). Transforming genes of human bladder and lung carcinoma cell lines are homologous to the ras genes of Harvey and Kirsten sarcoma viruses. *Proc. Natl Acad. Sci. USA*, **79**, 3637–3660.

ESRIG D, SPRUCK III CH, NICHOLS PW, CHAIWUN B, STEVEN K, GROSHEN S, CHEN S-C, SKINNER DG, JONES PA AND COTE RJ. (1993). p53 nuclear protein accumulation correlates with mutations in the p53 gene, tumor grade, and stage in bladder cancer. *Am. J. Pathol.*, **143**, 1389–1397.

ESRIG D, ELMAJIAN D, GROSHEN S, FREEMAN JA, STEIN JP, CHEN S-C, NICHOLS P, SKINNER DG, JONES PA AND COTE RJ. (1994). Accumulation of nuclear p53 and tumor progression in bladder cancer. *N. Engl. J. Med.*, **331**, 1259–1264.

FANNING P, BULOVAS K, SAINI KS, LIBERTINO JA, JOYCE AD AND SUMMERHAYES IC. (1992). Elevated expression of $pp60^{c-src}$ in low grade human bladder carcinomas. *Cancer Res.*, **52**, 1457–1462.

FRIXEN UH, BEHRENS J, SACHS M, EBERLE G, VOSS B, WARDA A, LOCHNER D AND BIRCHMEIER W. (1991). E-cadherin-mediated cell–cell invasiveness of human carcinoma cells. *J. Cell. Biol.*, **113**, 173–185.

FUJIMOTO K, YAMADA Y, OKAJIMA E, KAKIZOE T, SASAKI H, SUGIMURA T AND TERADA M. (1992). Frequent association of p53 gene mutation in invasive bladder cancer. *Cancer Res.*, **52**, 1393–1398.

FUJITA J, SRIVASTA SK, KRAUS MH, RHIM JS, TRONICK SR AND AARONSON SA. (1985). Frequency of molecular alterations affecting ras protooncogenes in human urinary tract tumours. *Proc. Natl Acad. Sci. USA*, **82**, 3849–3853.

GOODRICH DW, CHEN Y, SCULLY P AND LEE W-H. (1992). Expression of the retinoblastoma gene product in bladder carcinoma cells associates with a low frequency of tumor formation. *Cancer Res.*, **52**, 1968–1973.

HABUCHI T, KINOSHITA H, YAMADA H, KAKEHI Y, OGAWA O, WU W-J, TAKAHASHI R, SUGIYAMA T AND YOSHIDA O. (1994). Oncogene amplification in urothelial cancers with p53 gene mutation or MDM2 amplification. *J. Natl Cancer Inst.*, **86**, 1331–1335.

HOROWITZ JM, PARK S-H, BOGENMANN E, CHENG J-C, YANDELL DW, KAYE FJ, MINNA JD AND DRYJA TP. (1990). Frequent inactivation of the retinoblastoma anti-oncogene is restricted to a subset of human tumor cells. *Proc. Natl Acad. Sci. USA*, **87**, 2775–2779.

HOROWITZ J, YANDELL DW, PARK S-H, CANNING S, WHYTE P, BUCHKOVICH K, HARLOW E, WEINBERG RA AND DRYJA TP. (1989). Point mutational inactivation of the retinoblastoma antioncogene. *Science*, **243**, 937–940.

KAMB A, GRUIS NA, WEAVER-FELDHAUS J, LIU Q, HARSHMAN K, TAVTIGIAN SV, STOCKERT E, DAY III RS, JOHNSON BE AND SKOLNICK MH. (1994). A cell cycle regulator potentially involved in genesis of many tumor types. *Science*, **264**, 436–440.

KNOWLES MA AND WILLIAMSON M. (1993). Mutation of H-ras is infrequent in bladder cancer: confirmation by single-strand conformation polymorphism analysis, designed restriction fragment length polymorphisms, and direct seqeuncing. *Cancer Res.*, **53**, 133–139.

LEVESQUE P, RAMCHURREN N, SAINI K, JOYCE A, LIBERTINO J AND SUMMERHAYES IC. (1993). Screening of human bladder tumors and urine sediments for the presence of H-ras mutations. *Int. J. Cancer*, **55**, 785–790.

LIANES P, ORLOW I, ZHANG Z-F, OLIVA MR, SARKIS AS, REUTER VE AND CORDON-CARDO C. (1994). Altered patterns of MDM2 and TP53 expression in human bladder cancer. *J. Natl Cancer Inst.*, **86**, 1325–1330.

LOGOTHETIS CJ, XU H-J, RO JY, HU S-X, SAHIN A, ORDONEZ N AND BENEDICT WT. (1992). Altered expression of retinoblastoma protein and known prognostic variables in locally advanced bladder cancer. *J. Natl Cancer Inst.*, **84**, 1256–1261.

MORIYAMA M, AKIYAMA T, YAMAMOTO T, KAWAMOTO T, KATO T, SATO K, WATANUKI T, HIKAGE T, KATSUTA N AND MORI S. (1991). Expression of c-erbB-2 gene product in urinary bladder cancer. *J. Urol.*, **145**, 423–427.

MUNCASTER MM, COHEN BL, PHILLIPS RA AND GALLIE BL. (1992). Failure of Rb1 to reverse the malignant phenotype of human tumor cell lines. *Cancer Res.*, **52**, 654–661.

NEAL DE, BENNETT MK, HALL RB, MARSH C, ABEL PD, SAINSBURY JRC AND HARRIS AL. (1985). Epidermal-growth-factor receptors in human bladder cancer: comparison of invasive and superficial tumors. *Lancet*, **1**, 366–368.

NEAL DE, SHARPLES L, SMITH K, FENNELLY J, HALL RR AND HARRIS AL. (1990). The epidermal growth factor receptor and prognosis of bladder cancer. *Cancer*, **65**, 1619–1625.

PARADA LF, TABIN CJ, SHIH C AND WEINBERG RA. (1982). Human EJ bladder carcinoma oncogene is homologue of Harvey sarcoma virus ras gene. *Nature*, **297**, 474–478.

RUBINFELD B, SOUZA B, ALBERT I, MULLER O, CHAMBERLAIN SH, MASIARZ FR, MUNEMITSU S AND POLAKIS P. (1993). Association of the APC gene product with β-catenin. *Science*, **262**, 1731–1733.

SARKIS AS, DALBAGNI G, CORDON-CARDO C, ZHANG ZF, SHEINFELD J, FAIR WR, HERR HW AND REUTER VE. (1993). Nuclear overexpression of p53 protein in transitional cell bladder carcinoma: a marker for disease progression. *J. Natl Cancer Inst.*, **85**, 53–59.

SAUTER G, MOCH H, MOORE D, CARROLL P, KERSCHMANN R, CHEW K, MIHATSCH MK, GUDAT F AND WALDMAN F. (1993). Heterogeneity of erbB-2 gene amplification in bladder cancer. *Cancer Res.*, **53**, 2199–2203.

SIDRANSKY D, VON ESCHENBACH A, TSAI YC, JONES P, SUMMERHAYES I, MARSHALL F, PAUL M, GREEN P, HAMILTON SR, FROST P AND VOGELSTEIN B. (1991). Identification of p53 gene mutations in bladder cancer and urine samples. *Science*, **252**, 706–709.

SMITH KJ, JOHNSON KA, BRYAN TM, HILL DE, MARKOWITZ S, WILLSON JKV, PARASKEVA C, PETERSEN GM, HAMILTON SR, VOGELSTEIN B AND KINZLER KW. (1993). The APC gene product in normal and tumor cells. *Proc. Natl Acad. Sci. USA*, **90**, 2846–2850.

SOINI Y, TURPEENNIEMI-HUJANEN T, KAMEL D, AUTIO-HARMAINEN H, RISTELI J, RISTELI L, NUORVA K, PAAKKO P AND VAHAKANGAS K. (1993). p53 immunohistochemistry in transitional cell carcinoma and dysplasia of the urinary bladder correlates with disease progression. *Br. J. Cancer*, **68**, 1029–1035.

SPRUCK III CH, GONZALEZ-ZULUETA M, SHIBATA A, SIMONEAU AR, LIN M-F, GONZALES F, TSAI YC AND JONES PA. (1994). p16 gene in uncultured tumours. *Nature*, **370**, 183–184.

SU L-K, VOGELSTEIN B AND KINZLER KW. (1993). Association of the APC tumor suppressor protein with catenins. *Science*, **262**, 1734–1737.

TAKAHASHI R, HASHIMOTO T, XU H-J, HU S-X, MATSUI T, MIKI T, BIGO-MARSHAL H, AARONSON SA AND BENEDICT WF. (1991). The retinoblastoma gene functions as a growth and tumor suppressor in human bladder cells. *Proc. Natl Acad. Sci. USA*, **88**, 5257–5261.

VISVANATHAN KV, POCOCK RD AND SUMMERHAYES IC. (1988). Preferential and novel activation of H-ras in human bladder carcinomas. *Oncogene Res.*, **3**, 77–86.

WILLIAMSON MP, ELDER PA AND KNOWLES MA. (1994). The spectrum of TP53 mutations in bladder carcinomas. *Genes Chrom. Cancer*, **9**, 108–118.

WOOD DP, WARTINGER DD, REUTER V, CORDON-CARDO C, FAIR WR AND CHAGANTI RSK. (1991). DNA, RNA and immunohistochemical characterization of the HER-2/neu oncogene in transitional cell carcinoma of the bladder. *J. Urol.*, **146**, 1398–1401.

WRIGHT C, MELLON K, NEAL DE, JOHNSTON P, CORBETT IP AND HORNE CHW. (1990). Expression of c-*erb*B-2 protein product in bladder cancer. *Br. J. Cancer*, **62**, 764–767.

WRIGHT C, MELLON K, JOHNSTON P, LANE DP, HARRIS AL, HORNE CHW AND NEAL DE. (1991). Expression of mutant p53, c-*erb*B-2 and the epidermal growth factor receptor in transitional cell carcinoma of the human urinary bladder. *Br. J. Cancer*, **63**, 967–970.